# Supplementary figures and images for: Novel insights into RAGE signaling pathways during the progression of amyotrophic lateral sclerosis in RAGE-deficient SOD1 G93A mice
Source: PLoS One. 2024 Mar 8;19(3):e0299567. doi: 10.1371/journal.pone.0299567 (PMC10923448; doi:10.1371/journal.pone.0299567)

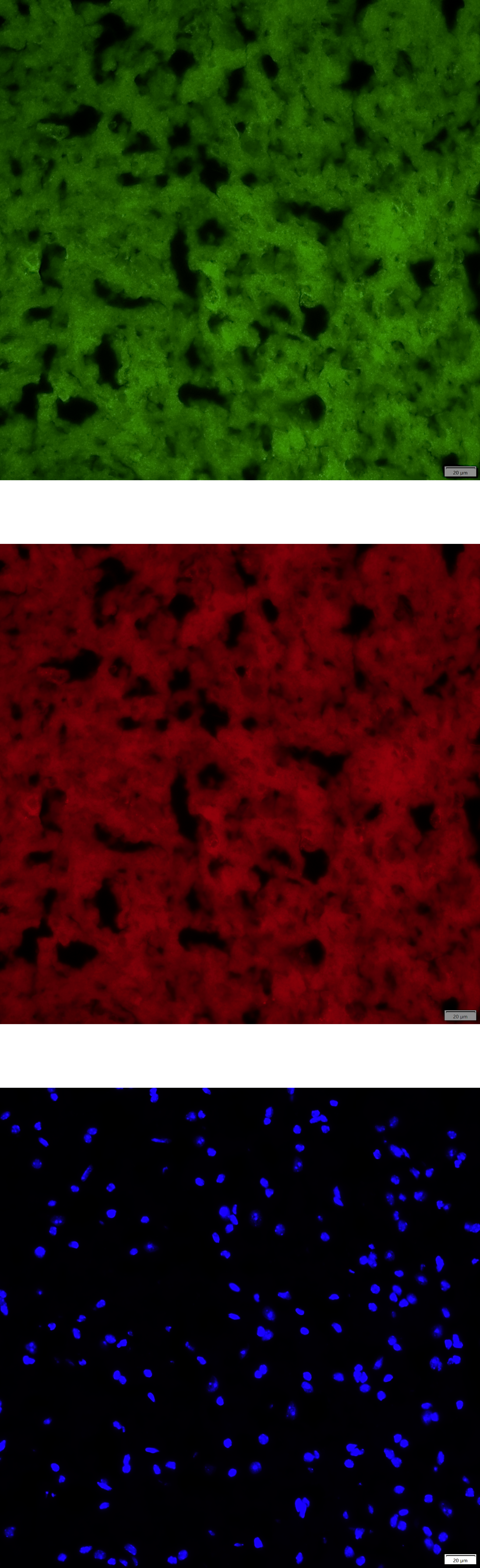

Supplement: S1 Fig — No primary antibodies, only secondary antibodies were used (Table 2). Images were taken under 20× objective with 0.7 numerical aperture (20× /0.7). Scale bar = 20 μm. (TIF) [file pone.0299567.s001.tif]
