# Supplementary material for: Novel insights into RAGE signaling pathways during the progression of amyotrophic lateral sclerosis in RAGE-deficient SOD1 G93A mice
Source: PLoS One. 2024 Mar 8;19(3):e0299567. doi: 10.1371/journal.pone.0299567 (PMC10923448; doi:10.1371/journal.pone.0299567)
Supplement: S1 Raw images — (PDF) [file pone.0299567.s002.pdf]

S 1 raw images

Western blot raw data.

Uncropped blots for western blot panels presented in main figures. Western blots and densitometry analysis of proteins in spinal cord. An equal amount of protein (40 µg) was fractionated on 15-well 4–15% Mini-PROTEAN® TGX™ Precast Protein Gels (Bio-Rad, CA, USA) and transferred to nitrocellulose membranes. The bands were visualized with ChemiDoc Imag-ing Systems (Bio-Rad); automatic exposures. Images were quantified densitometrically with ImageJ Software 1.50i (Wayne Rasband, MD, USA) and compared to experimental condition after normalization to the total amount of protein in a sample. In some cases, the membrane has been cut to allow for more analyzes. Figure panels are indicated above the corresponding western blot set. Loading controls and the respective experimental samples were run on the same blots. M – marker, S100B – S100 calcium-binding protein B, HMGB1 – High Mobility Group Box 1, CML – N(epsilon)-(carboxymethyl)lysine, ACTB – beta actin.

Full gel image annotation for the western blot. The ladder is shown as M. **S100B protein**. WT: 1-3, SOD1:4-6, RAGE KO SOD1 90: 7-10, RAGE KO SOD1 T: 11-14.

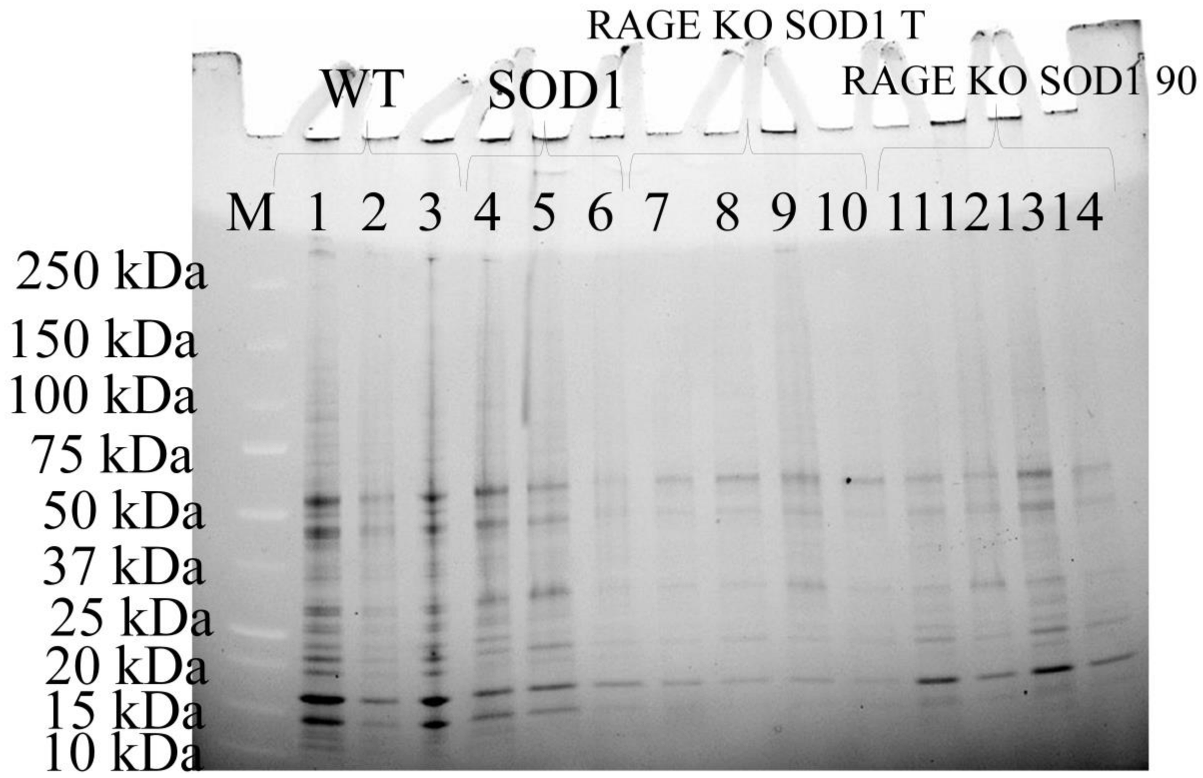
RAGE KO SOD1 T
WT
SOD1
RAGE KO SOD1 90
M 1 2 3 4 5 6 7 8 9 10 11 12 13 14
250 kDa
150 kDa
100 kDa
75 kDa
50 kDa
37 kDa
25 kDa
20 kDa
15 kDa
10 kDa

Full gel image annotation for the western blot. The ladder is shown as M. **HMGB1, CML, ACTB proteins**. WT: 1-3, SOD1:4-6, RAGE KO SOD1 90: 7-10, RAGE KO SOD1 T: 11-14.

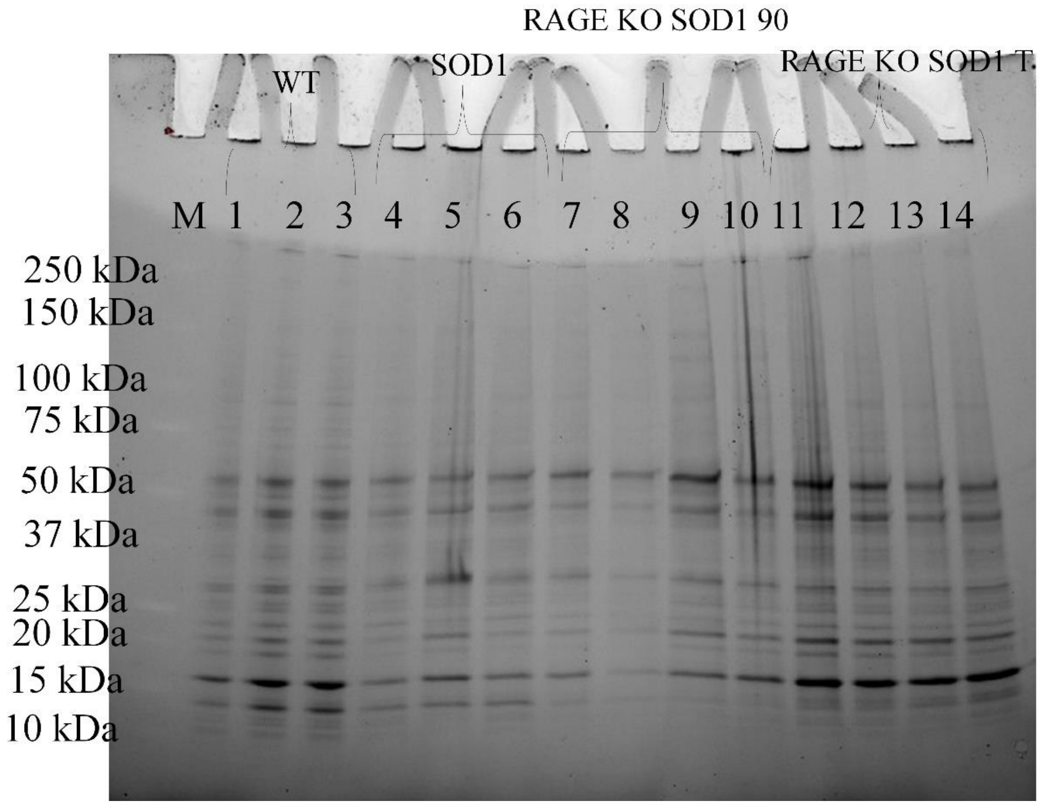

Full blot image annotation for the western blot shown in Figs 3-4. The ladder is shown as M. **HMGB1, S100B, CML** proteins. WT: 1-3, SOD1:4-6, RAGE KO SOD1 90: 7-10, RAGE KO SOD1 T: 11-14.

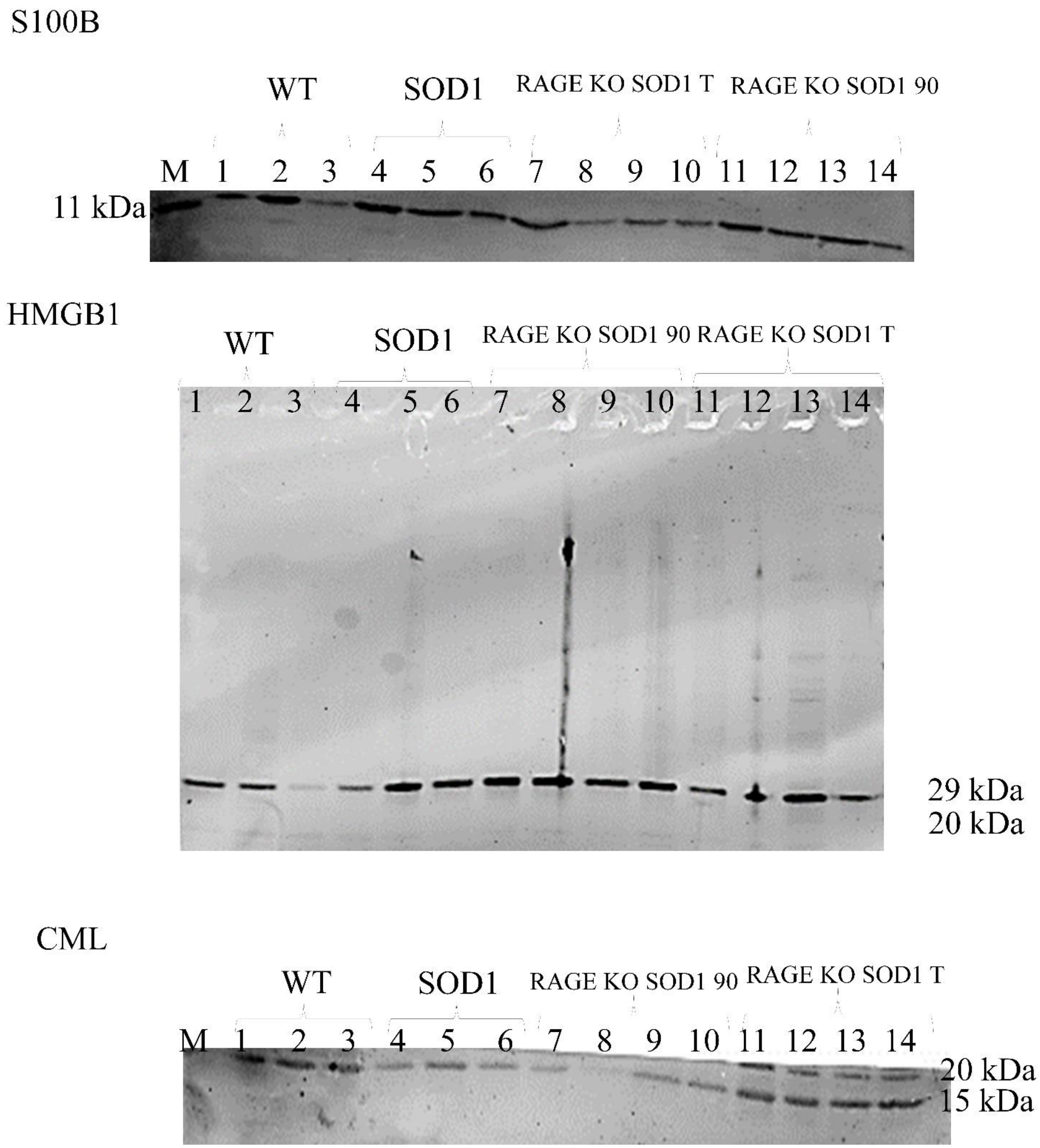
S100B
WT
SOD1
RAGE KO SOD1 T
RAGE KO SOD1 90
M 1 2 3 4 5 6 7 8 9 10 11 12 13 14
11 kDa
HMGB1
WT
SOD1
RAGE KO SOD1 90
RAGE KO SOD1 T
1 2 3 4 5 6 7 8 9 10 11 12 13 14
29 kDa
20 kDa
CML
WT
SOD1
RAGE KO SOD1 90
RAGE KO SOD1 T
M 1 2 3 4 5 6 7 8 9 10 11 12 13 14
20 kDa
15 kDa

Full blot image annotation for the western blot shown in Figs 3-4. The ladder is shown as M. **ACTB protein**. WT: 1-3, SOD1:4-6, RAGE KO SOD1 90: 7-10, RAGE KO SOD1 T: 11-14.

ACTB

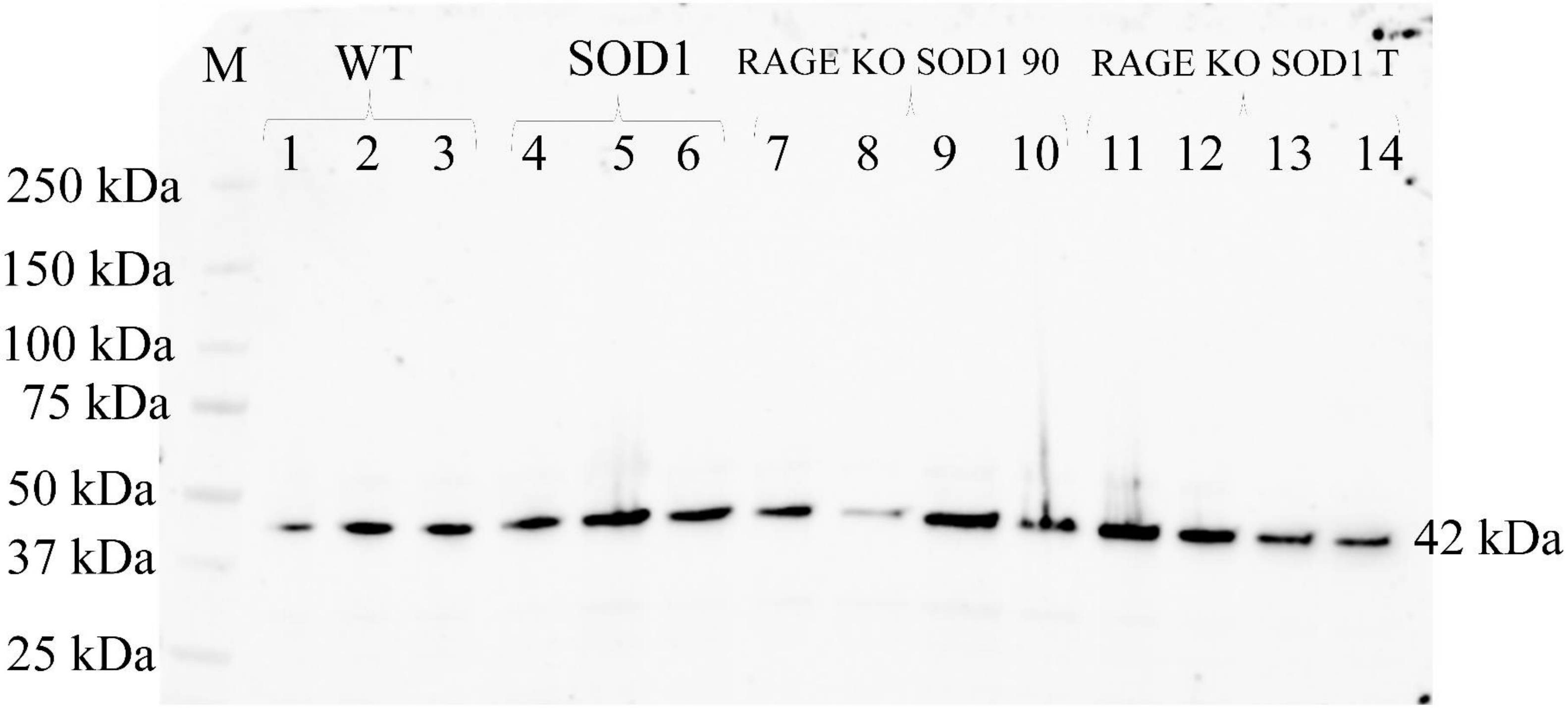

Table of western blot raw data

Images were quantified densitometrically with ImageJ Software 1.50i (Wayne Rasband, MD, USA) and compared to experimental condition after normalization to the total amount of protein in a sample. **S100B – S100 calcium-binding protein B, HMGB1 – High Mobility Group Box 1, CML – N(epsilon)-(carboxymethyl)lysine, ACTB – beta actin**.

Test protein	Number of sample WT	WT – value (test protein/total protein)	Number of sample SOD1	SOD1 – value (test protein/total protein)	Number of sample RAGE KO SOD1 90	RAGE KO SOD1 90 – value (test protein/total protein)	Number of sample RAGE KO SOD1 T	RAGE KO SOD1 T – value (test protein/total protein)
S100B	1	2865,593	4	1743,396	11	5972,642	7	1816,853
	2	1156,238	5	1442,581	12	3972,677	8	1236,862
	3	1955,205	6	1230,508	13	3514,622	9	3716,42
	-	-	-		14	3179,694	10	818,72
HMGB1	1	1875,766	4	2150,589	7	6811,776	11	2077,014
	2	3554,832	5	6482,724	8	10790,58	12	1401,837
	3	3304,165	6	2832,659	9	14342,63	13	369,3649
	-		-		10	10391,39	14	876,2425
CML	1	6723,525	4	6723,525	7	988,2098	11	1271,439
	2	2354,956	5	2354,956	8	551,7919	12	1227,773
	3	2179,703	6	2179,703	9	1158,051	13	1256,112
	-		-		10	1075,85	14	1375,789
ACTB	1	24,6819	4	90,07150168	7	91,59793	11	141,7777356
	2	93,69039495	5	206,8088748	8	25,07574	12	113,8714835
	3	70,43845185	6	125,6229226	9	184,0765	13	52,06216351
	-		-	-	10	106,9381	14	38,14340101
